# Supplementary material for: TLR7 mediated viral recognition results in focal type I interferon secretion by dendritic cells
Source: Nat Commun. 2017 Nov 17;8:1592. doi: 10.1038/s41467-017-01687-x (PMC5693993; doi:10.1038/s41467-017-01687-x)
Supplement: Supplementary file 1 — Supplementary Information [file 41467_2017_1687_MOESM1_ESM.pdf]

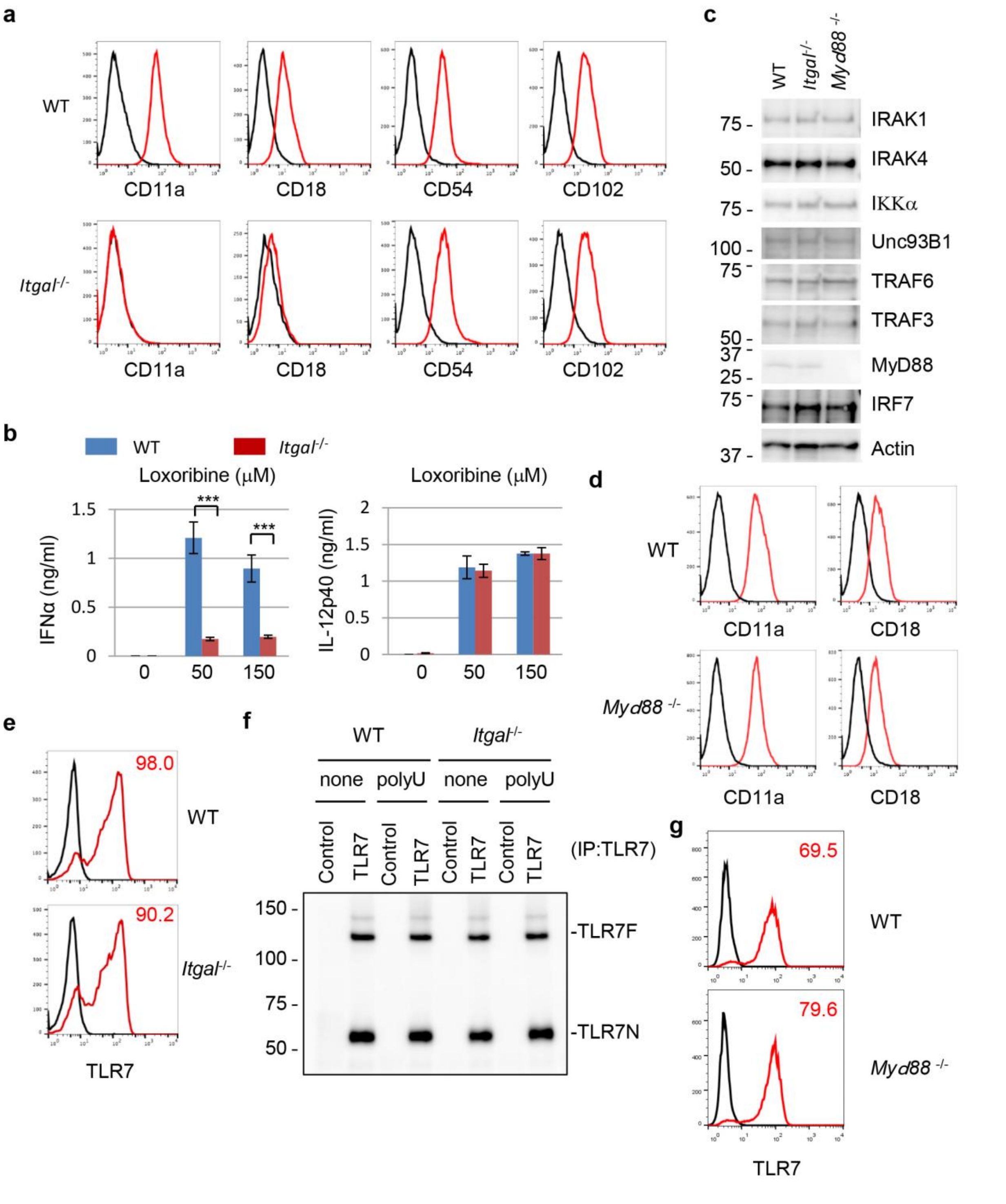

Supplementary Figure 1. Impaired IFN-α production in *Itgal*$^{-/-}$ BM-pDCs

(**a**) WT and *Itgal*$^{-/-}$ BM-pDCs were stained with Abs to indicated molecules. Red and black histograms show staining with primary Ab to indicated molecules or isotype-matched control Ab,

respectively. (**b**) BM-pDCs were stimulated or not with loxoribine at the indicated concentrations for 24h prior to ELISA determination of IFNα and IL-12p40 expression levels. Data shown are mean ± s.d. from triplicate wells. The experiments were repeated 3 times. ***P< 0.001 (Unpaired two-tailed t-test). (**c**) Shown is immunostaining of the indicated signaling molecules in whole cell lysates from WT, *Itgal*$^{-/-}$, and *Myd88*$^{-/-}$ BM-pDCs. (**d**) WT and *Myd88*$^{-/-}$ BM-pDCs were stained with Abs to indicated molecules. Red and black histograms show staining with primary Ab to indicated molecules or isotype-matched control Ab, respectively. (**e**, **g**) Red histograms show membrane-permeabilized staining of TLR7 in WT or *Itgal*$^{-/-}$ or *Myd88*$^{-/-}$ BM-pDCs (black histograms indicate staining with isotype-matched control Ab). Mean fluorescence intensities of TLR7 staining are shown in the panels. (**f**) WT or *Itgal*$^{-/-}$ BM-pDCs were stimulated or not with 25 μg/ml polyU for 1.5h, and then subjected to lysis and immunoprecipitation using Abs against TLR7 or an isotype-matched control. Shown is immunostaining of both the uncleaved full-length TLR7 (TLR7F) and its cleaved N-terminal fragment (TLR7N).

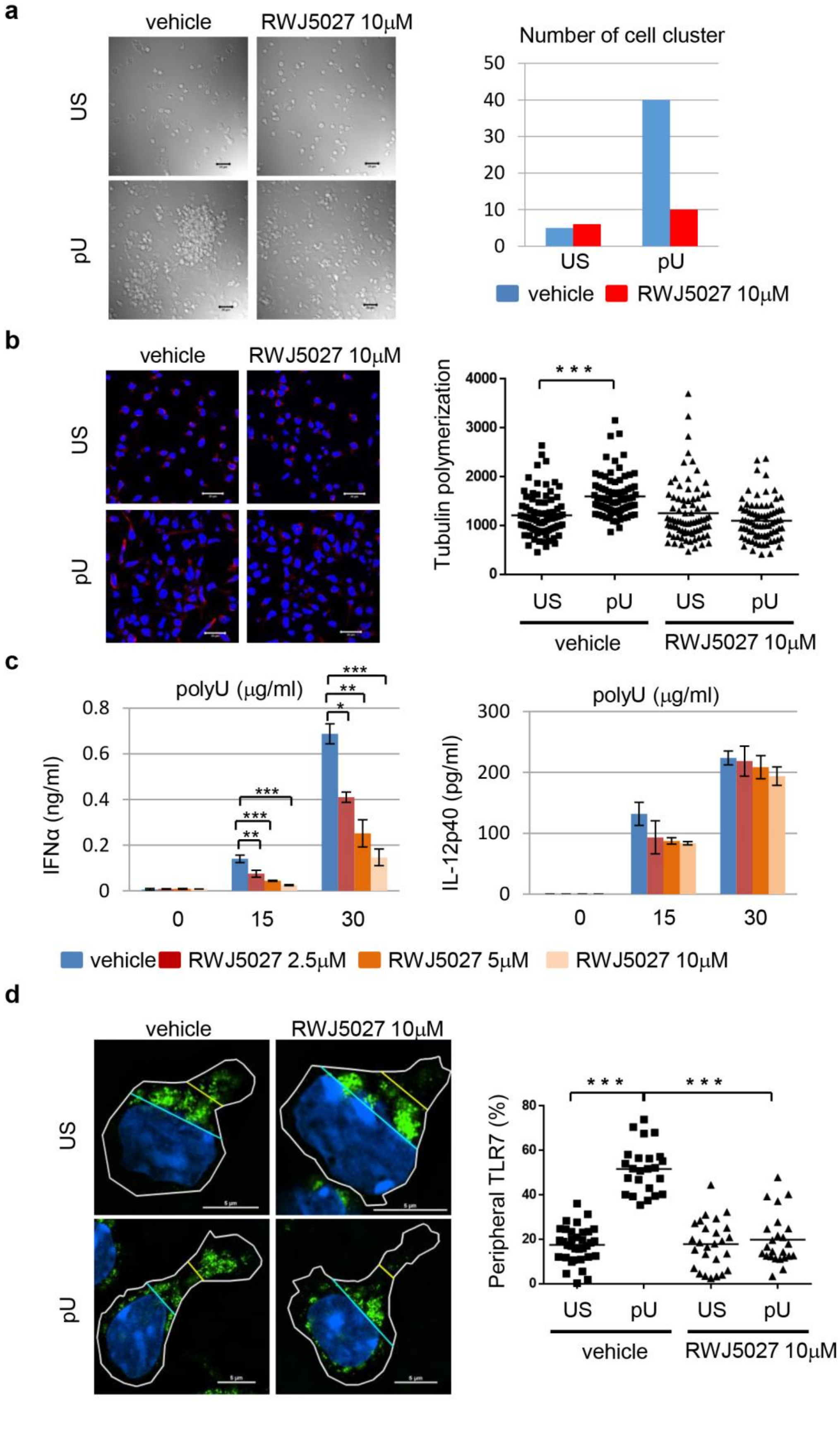
a
vehicle
RWJ5027 10μM
US
pU
Number of cell cluster
50
40
30
20
10
0
US
pU
vehicle
RWJ5027 10μM
b
vehicle
RWJ5027 10μM
US
pU
Tubulin polymerization
4000
3000
2000
1000
0
***
US
pU
US
pU
vehicle
RWJ5027 10μM
c
polyU (μg/ml)
0.8
0.6
0.4
0.2
0
IFNα (ng/ml)
***
**
*
***
***
**
0
15
30
polyU (μg/ml)
300
200
100
0
IL-12p40 (pg/ml)
0
15
30
vehicle
RWJ5027 2.5μM
RWJ5027 5μM
RWJ5027 10μM
d
vehicle
RWJ5027 10μM
US
pU
Peripheral TLR7 (%)
80
60
40
20
0
***
***
US
pU
US
pU
vehicle
RWJ5027 10μM

Supplementary Figure 2. An inhibitor of LFA-1/ICAM association significantly suppressed pDC clustering, TLR7 trafficking, and IFN-α production

(**a, b, d**) WT BM-pDCs were pretreated with the inhibitor of LFA-1/ICAM binding, RWJ5027, at 10μM for 1h, and then left unstimulated (US) or activated (pU) with polyU at 25 μg/ml for 4h (**a, b**) or 3h (**d**). (**a**) Clustering of pDCs was visualized by microscopy and counted in 25 visual areas. Scale bar, 20μm. (**b**) α-tubulin was stained and fluorescence intensities of α-tubulin staining in each cell were measured for statistical analysis (n>80). Scale bar, 20μm. (**c**) BM-pDCs were pretreated with the LFA-1/ICAM binding inhibitor, RWJ50271, for 1 h at the indicated concentration and stimulated with polyU at 15 or 30 μg/ml for 24h. Production of IFNα and IL-12 p40 was measured by ELISA. Data shown are mean ± s.d. from triplicate wells. The experiments were repeated 3 times with similar results. (**d**) After permeabilization, BM-pDCs were stained with Abs against TLR7 (green) together with DAPI staining of cell nuclei (blue) prior to analysis by SIM. Plasma membranes is indicated by white line. Blue lines are placed at the boundary between cytoplasm and nucleus. Yellow lines are at the middle of blue lines and the tip of the polarized cytoplasm. These lines were used for quantification of TLR7 trafficking. Right panel shows quantification of peripheral TLR7 as a percentage of total staining in perinuclear and peripheral regions in each cell (n>23). Scale bar, 5μm. ****P*<0.001, ***P*<0.01, **P*<0.05 (Unpaired two-tailed t-test)

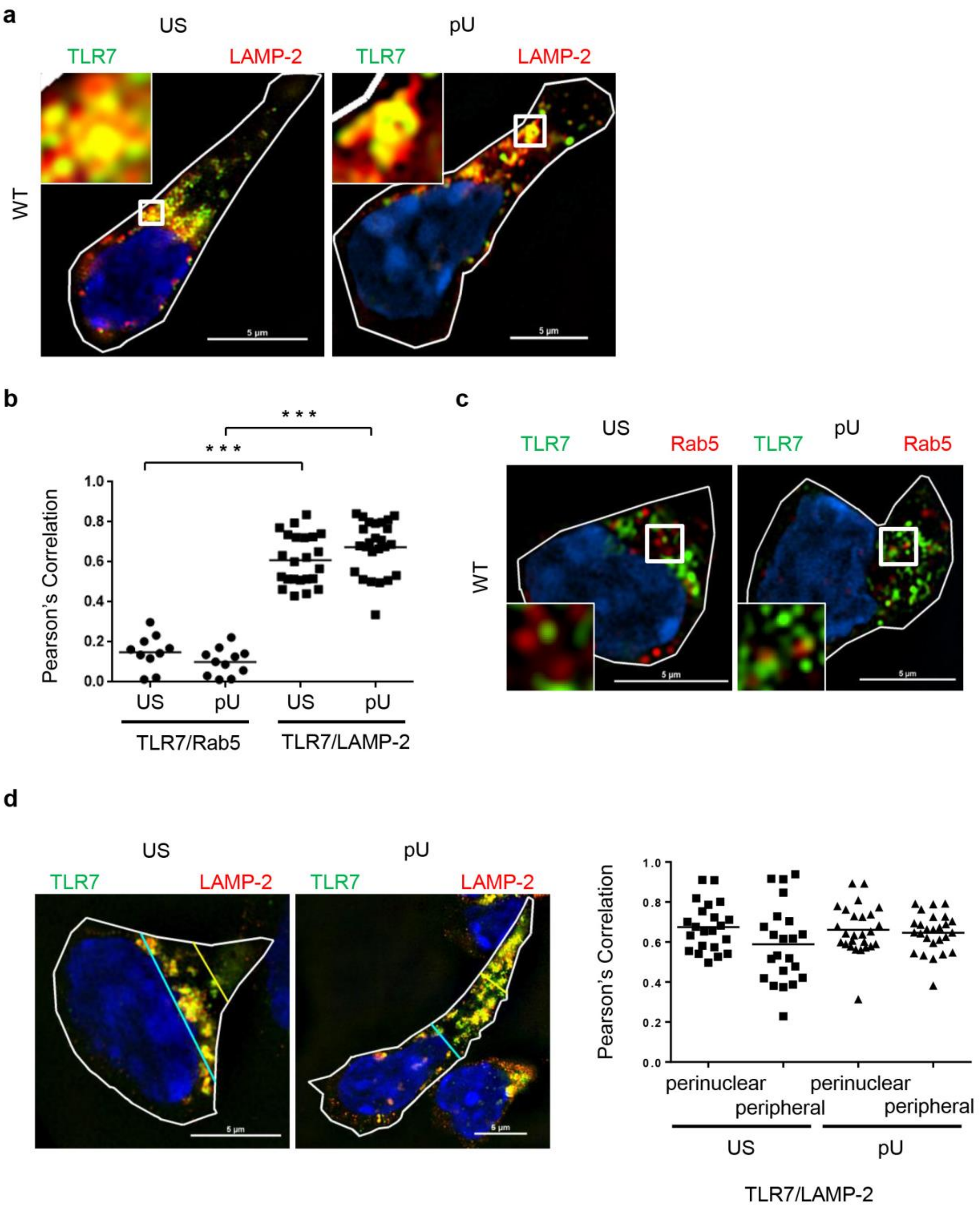

Supplementary Figure 3. TLR7 localization in lysosomes.

(**a-d**) BM-pDCs were either left unstimulated (US) or activated (pU) with 25 µg/ml polyU for 3h. pDCs were stained with Abs against TLR7, LAMP-2, and Rab5 as indicated. Nuclei were

visualized via DAPI staining (blue) (**a**, **c**). Higher magnification images of boxed regions are shown in the insets. (**b**) Statistical analyses of TLR7 co-localization with LAMP-2 (**a**) or Rab5 (**c**) are shown (n>9). (**d**) To divide perinuclear region and peripheral region, blue lines and yellow lines were drawn. Blue lines are placed at the boundary between cytoplasm and nucleus. Yellow lines are at the middle of blue lines and the tip of the polarized cytoplasm. Right panel shows statistical analyses of TLR7 co-localization with LAMP-2 in perinuclear and peripheral region (n>21). Scale bar, 5μm. *** $P< 0.001$ (Unpaired two-tailed t-test)

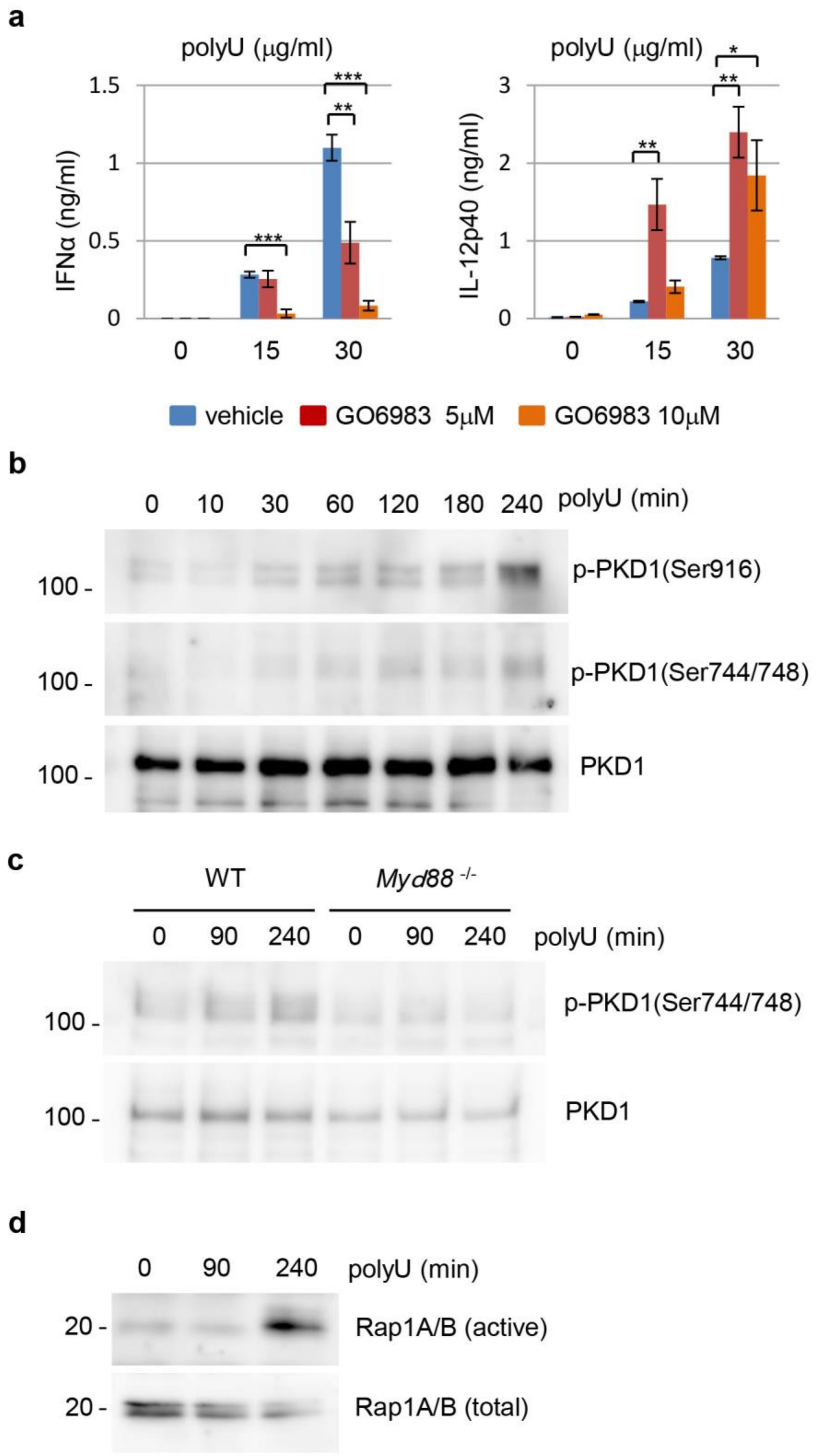

Supplementary Figure 4. Inside-out signaling for LFA-1 activation is essential for IFN-α expression

(**a**) WT BM-pDCs were pretreated with the indicated concentration of the Protein Kinase C inhibitor Go6983 for 1h, and then activated with polyU at 15 or 30 μg/ml for 24h. Production of IFNα and IL-12 p40 was measured by ELISA. Data shown are mean ± s.d. from triplicate wells. *$P < 0.05$, **$P < 0.01$, *** $P < 0.001$ (Unpaired two-tailed t-test). The experiments were repeated 3 times with similar results. (**b, c, d**) WT or *Myd88*$^{-/-}$ BM-pDCs were stimulated with 25 μg/ml polyU for the indicated time. Immunostaining of the phosphorylated PKD1 and PKD1 in whole cell lysate is shown (**b, c**). Lysates were mixed with RalGDS Rap binding domain agarose beads for pull-down assay, and then immunostaining with anti-Rap1 Ab. Immunostaining of Rap1 in whole cell lysate is also shown in the lower panel (**d**).

a

Gene symbol	Peptide-spectrum match: GFP	Peptide-spectrum match: Unc93B1	SAINT: Probability score: Unc93B1
Unc93b1	0	20	1
Tlr7	0	44	1
Tlr13	0	41	1
Tlr8	0	9	0.94
Arl8a	1	8	0.9
Arl8b	1	11	0.97

b

	Peptide-spectrum match			
	control		anti-TLR7	
	Tlr7$^{-/-}$	WT	*Tlr7*$^{-/-}$	WT
Arl8a/Arl8b shared	5	5	5	79
Arl8a unique	0	0	0	36
Arl8b unique	0	0	0	37

c

```
Score =  363 bits (933),  Expect = 1e-130, Method: Compositional matrix adjust.
 Identities = 170/186 (91%), Positives = 183/186 (98%), Gaps = 0/186 (0%)

Arl8a  1    MIALFNKLLDWFKALFWKEEMELTLVGLQYSGKTTFVNVIASGQFNEDMIPTVGFNMRKI  60
            M+AL ++LLDWF++LFWKEEMELTLVGLQYSGKTTFVNVIASGQF+EDMIPTVGFNMRK+
Arl8b  1    MLALISRLLDWFRSLFWKEEMELTLVGLQYSGKTTFVNVIASGQFSEDMIPTVGFNMRKV  60

Arl8a  61   TKGNVTIKLWDIGGQPRFRSMWERYCRGVSAIVYMVDAADQEKIEASKNELHNLLDKPQL  120
            TKGNVTIK+WDIGGQPRFRSMWERYCRGV+AIVYM+DAAD+EKIEAS+NELHNLLDKPQL
Arl8b  61   TKGNVTIKIWDIGGQPRFRSMWERYCRGVNAIVYMIDAADREKIEASRNELHNLLDKPQL  120

Arl8a  121  QGIPVLVLGNKRDLAGALDEKELIEKMNLSAIQDREICCYSISCKEKDNIDITLQWLIQH  180
            QGIPVLVLGNKRDL  ALDEK+LIEKMNLSAIQDREICCYSISCKEKDNIDITLQWLIQH
Arl8b  121  QGIPVLVLGNKRDLPNALDEKQLIEKMNLSAIQDREICCYSISCKEKDNIDITLQWLIQH  180

Arl8a  181  SKSRRS  186
            SKSRRS
Arl8b  181  SKSRRS  186
```

d

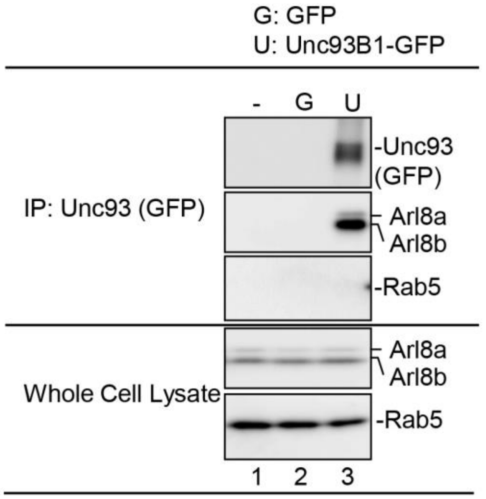

e

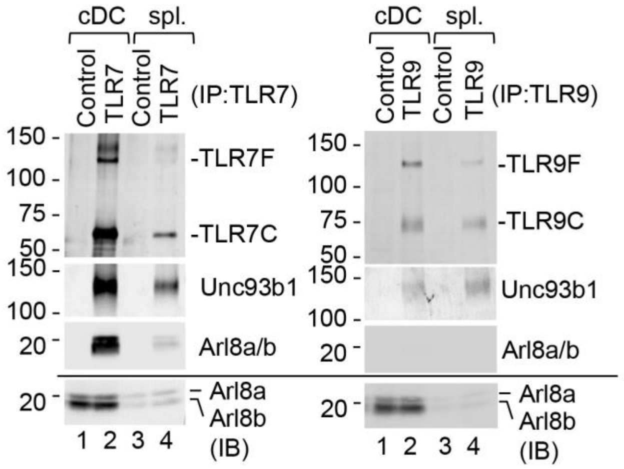

Supplementary Figure 5. Association of TLR7 with Arl8a and Arl8b

(**a**) Flag-GFP or wild-type (WT) Unc93B1-Flag-GFP was expressed in bone marrow-derived conventional dendritic cells (BM-cDCs) and immunoprecipitated with anti-Flag Ab. Interacting molecules were identified by LC-MS/MS. Associated proteins identified with high confidence (SAINT probability score ≥ 0.9) were then listed with peptide-spectrum matches (PSMs). (**b**) WT or *Tlr7*$^{-/-}$ BM-Macrophages were lysed and immunoprecipitated with Abs against TLR7 or IgG isotype-matched control prior to analysis by LC-MS/MS. (**c**) Comparison of amino acid sequences of Arl8a and Arl8b using protein-protein BLAST, where (+) indicates a positive match. Squares represent identified as shared (red), Arl8a-unique (blue), and Arl8b-unique (green) peptide sequences in TLR7 immunoprecipitation experiment. Since leucine (L) and isoleucine (I) have the same mass, L and I were regarded as identical. (**d**) Macrophage cell line RAW264.7 (lane 1), RAW264.7 cells expressing GFP (lane 2), and RAW264.7 cells expressing Unc93B1-GFP (lane 3) were subjected to immunoprecipitation with anti-GFP mAb followed by immunostaining with Abs against GFP, Arl8, or Rab5 (upper 3 panels). Immunostaining of whole cell lysate with Abs against Arl8 or Rab5 is also shown (lower 2 panels). (**e**) BM-cDCs or total spleen cells were lysed and immunoprecipitated using Abs against TLR7 or TLR9 (or isotype-matched irrelevant mAb; lanes 1 and 3) before probing the precipitate for the presence of TLR7, TLR9, Unc93B1, and Arl8a/b. Both the full-length form of the receptor (TLR7/9F) and cleaved C-terminal fragment (TLR7/9C) were detected. Bottom panel shows immunoprobing of cell lysate for the presence of Arl8a/b.

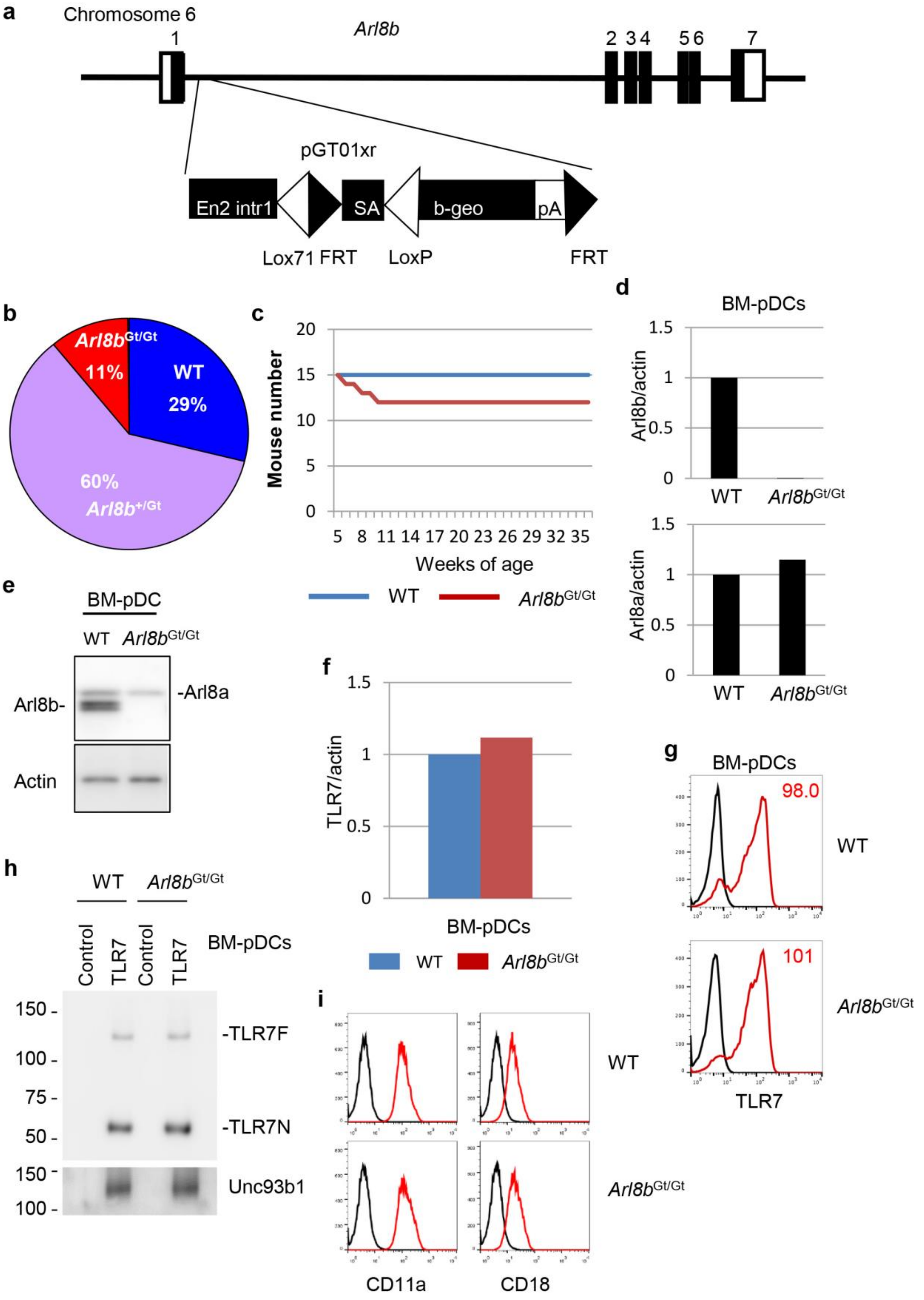
a
Chromosome 6
Arl8b
1
2 3 4
5 6
7
pGT01xr
En2 intr1
SA
b-geo
pA
Lox71 FRT
LoxP
FRT
b
Arl8bGt/Gt
11%
WT
29%
60%
Arl8b+/Gt
c
Mouse number
Weeks of age
WT
Arl8bGt/Gt
d
BM-pDCs
Arl8b/actin
Arl8a/actin
WT
Arl8bGt/Gt
e
BM-pDC
WT
Arl8bGt/Gt
Arl8b-
-Arl8a
Actin
f
TLR7/actin
BM-pDCs
WT
Arl8bGt/Gt
g
BM-pDCs
98.0
WT
101
Arl8bGt/Gt
TLR7
h
WT
Arl8bGt/Gt
Control
TLR7
Control
TLR7
BM-pDCs
-TLR7F
-TLR7N
Unc93b1
i
WT
Arl8bGt/Gt
CD11a
CD18

Supplementary Figure 6. Expression of Arl8b and TLR7 in *Arl8b*$^{Gt/Gt}$ mice

(**a**) Genomic configuration of the *Arl8b* locus in *Arl8b*$^{Gt/Gt}$ mice. Detailed information on the gene-trapped allele is available at the MGI Web site (MGI ID:3852843). (**b**) Genotypes of the total 139 offspring obtained from *Arl8b*$^{Gt/+}$ parents. (**c**) Survival of wild-type (blue) and *Arl8b*$^{Gt/Gt}$ mice (red) are shown. (**d**) Real-time PCR detection of Arl8b mRNA (upper panel) or Arl8a mRNA (lower panel) was conducted using cDNA derived from either WT or *Arl8b*$^{Gt/Gt}$ BM-pDCs (data are normalized to actin mRNA levels). (**e**) Cell lysates were prepared from WT or *Arl8b*$^{Gt/Gt}$ BM-pDCs prior to immunostaining for Arl8a/b and actin. (**f**) Real-time PCR detection of TLR7 mRNA in cDNA derived from WT (blue) or *Arl8b*$^{Gt/Gt}$ (red) BM-pDCs (data are normalized to actin mRNA). (**g**) TLR7 protein expression in WT (upper) or *Arl8b*$^{Gt/Gt}$ (lower) BM-pDCs as assessed by flow cytometry. Histograms show staining of membrane-permeabilized cells with anti-TLR7 mAb (red trace) or IgG isotype-matched control (black trace). Inset numbers indicate mean fluorescence intensity of TLR7 staining. (**h**) WT or *Arl8b*$^{Gt/Gt}$ BM-pDCs were immunoprecipitated using Abs against TLR7 (or IgG isotype-matched control) and the precipitate was probed for the presence of TLR7 (upper blot) and Unc93B1 (lower blot). TLR7F, uncleaved full-length form. TLR7N, cleaved N-terminal fragment. (**i**) WT and *Arl8b*$^{Gt/Gt}$ BM-pDCs were stained with Abs to CD11a or CD18. Red and black histograms show staining with primary Ab to indicated molecules or isotype-matched control Ab, respectively.

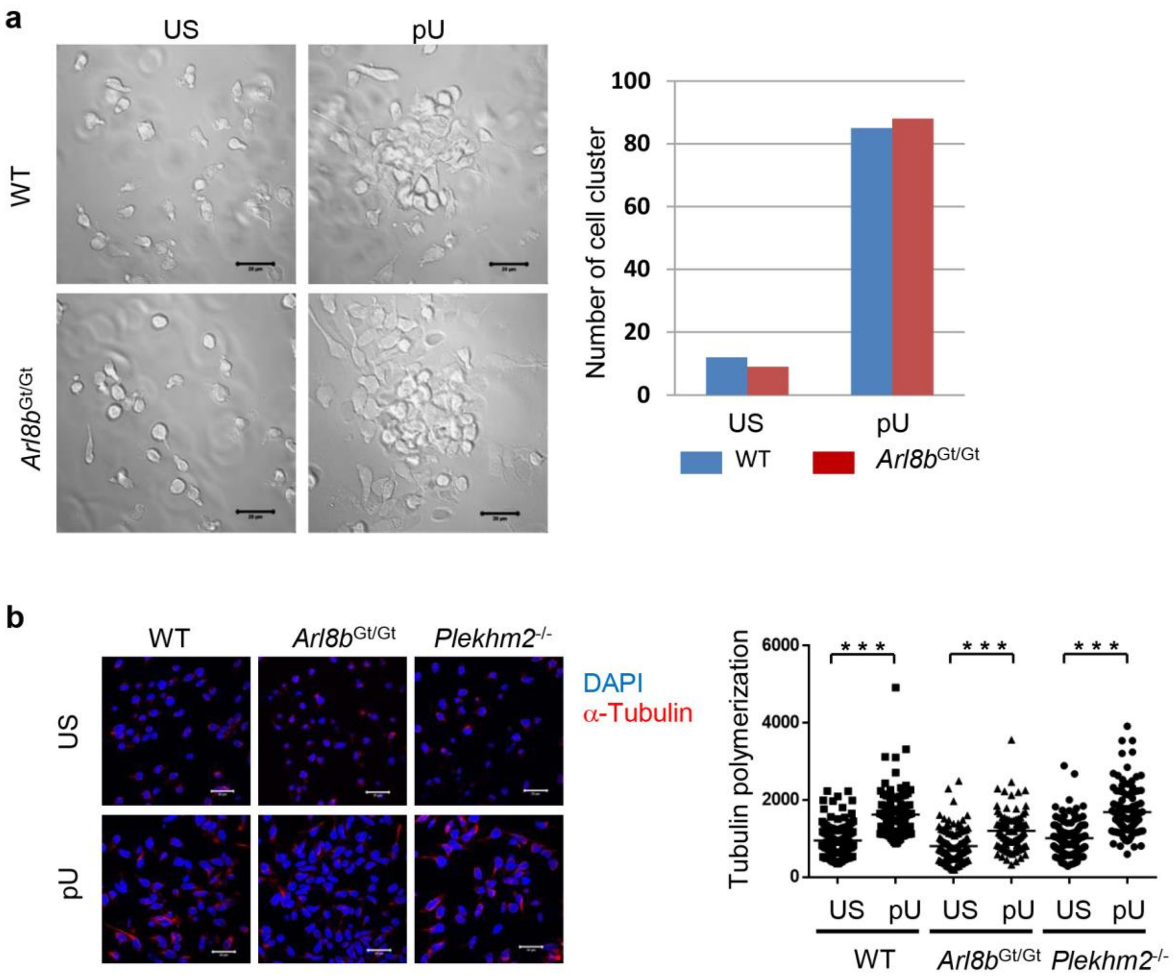

Supplementary Figure 7. Clustering and TLR7 responses in *Arl8b*$^{Gt/Gt}$ and *Plekhm2*$^{-/-}$ BM-pDCs.

(**a**) WT, and *Arl8b*$^{Gt/Gt}$ BM-pDCs were stimulated (pU) or not (US) with polyU at 25 µg/ml for 4h. Clustering of pDCs was visualized by microscopy and counted in 40 visual areas for statistical analyses. Scale bar, 20µm. (**b**) WT, *Arl8b*$^{Gt/Gt}$, and *Plekhm2*$^{-/-}$ BM-pDCs were left unstimulated (US) or stimulated (pU) with polyU at 25 µg/ml for 4h. α-tubulin (red) was stained and fluorescence intensities of α-tubulin staining in each cell were measured and shown as a dot for statistical analysis (n>95). ***P< 0.001 (Unpaired two-tailed t-test)

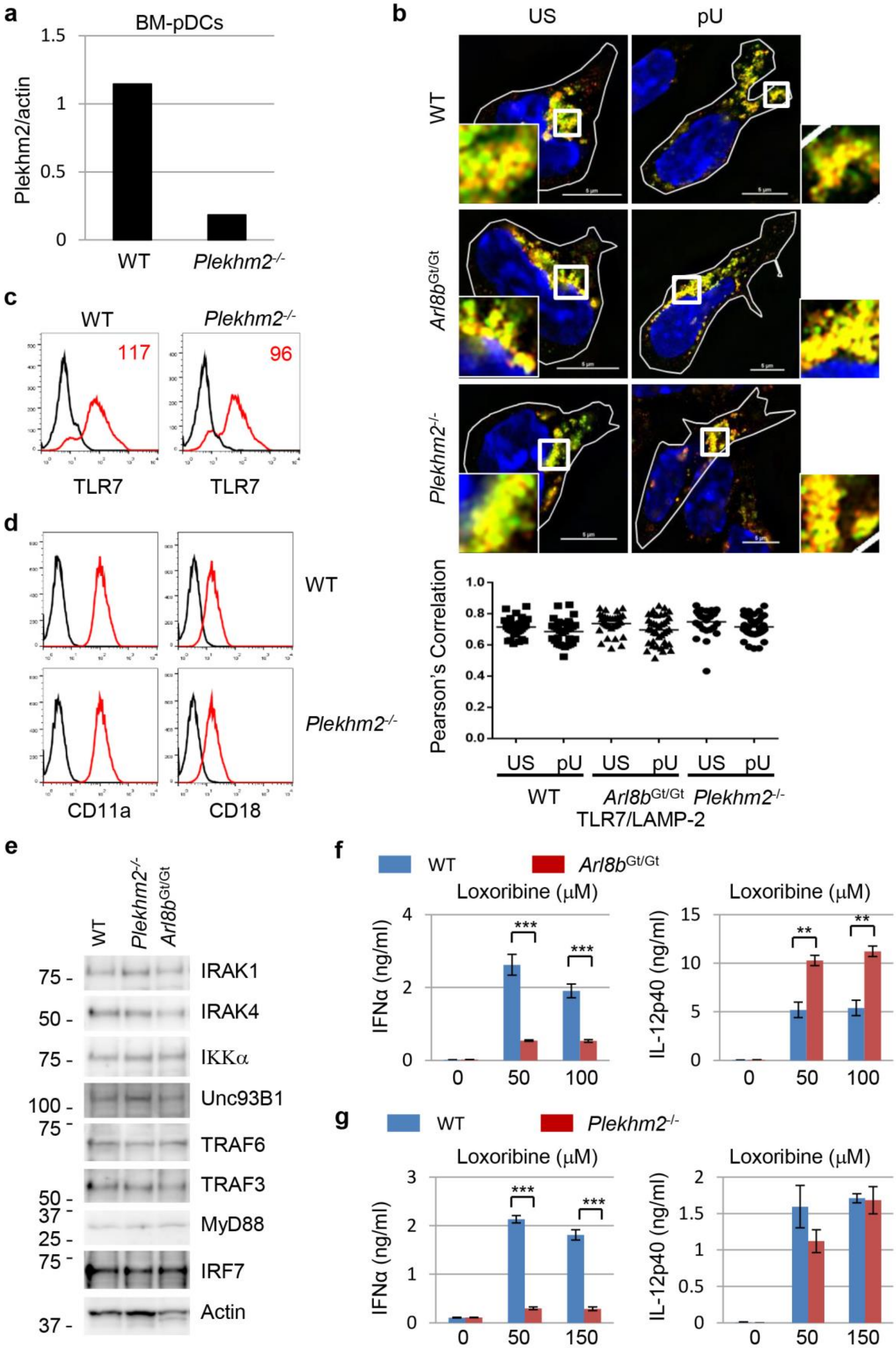
a
BM-pDCs
Plekhm2/actin
WT
Plekhm2-/-
b
US
pU
WT
Arl8bGt/Gt
Plekhm2-/-
Pearson's Correlation
US pU US pU US pU
WT Arl8bGt/Gt Plekhm2-/-
TLR7/LAMP-2
c
WT
Plekhm2-/-
117
96
TLR7
TLR7
d
WT
Plekhm2-/-
CD11a
CD18
e
WT
Plekhm2-/-
Arl8bGt/Gt
IRAK1
IRAK4
IKKα
Unc93B1
TRAF6
TRAF3
MyD88
IRF7
Actin
f
WT
Arl8bGt/Gt
Loxoribine (μM)
IFNα (ng/ml)
IL-12p40 (ng/ml)
g
WT
Plekhm2-/-
Loxoribine (μM)
IFNα (ng/ml)
IL-12p40 (ng/ml)

Supplementary Figure 8. TLR7 responses and colocalization of TLR7 and Lamp2 in *Arl8b*$^{Gt/Gt}$ and *Plekhm2*$^{-/-}$ BM-pDCs.

(**a**) Real-time PCR detection of SKIP mRNA using cDNA derived from WT or *Plekhm2*$^{-/-}$ BM-pDCs (data are normalized to actin mRNA). (**b**) WT, *Arl8b*$^{Gt/Gt}$, and *Plekhm2*$^{-/-}$ BM-pDCs were either left unstimulated (US) or activated (pU) with 25 µg/ml polyU for 3h. pDCs were stained with Abs against TLR7 (green), and LAMP-2 (red) as indicated. Nuclei were visualized via DAPI staining (blue). Inlets show higher magnification images of the region indicated by squares. The bottom panel shows statistical analyses of TLR7 co-localization with LAMP-2 in each cell (n>26). (**c**) TLR7 protein expression in WT (upper) or *Plekhm2*$^{-/-}$ (lower) BM-pDCs as assessed by flow cytometry. Histograms show staining of membrane-permeabilized cells with anti-TLR7 mAb (red trace) or IgG isotype-matched control (black trace). Inset numbers indicate mean fluorescence intensity of TLR7 staining. (**d**) WT and *Plekhm2*$^{-/-}$ BM-pDCs were stained with Abs to CD11a or CD18. Red and black histograms show staining with primary Ab to indicated molecules or isotype-matched control Ab, respectively. (**e**) Immunostaining of the indicated signaling molecules in whole cell lysates from WT, *Arl8b*$^{Gt/Gt}$, and *Plekhm2*$^{-/-}$ BM-pDCs is shown. (**f**, **g**) WT, *Arl8b*$^{Gt/Gt}$, and *Plekhm2*$^{-/-}$ BM-pDCs were either left unstimulated or activated with loxoribine at the indicated concentrations for 24h before determination of IFNα and IL-12p40 expression by ELISA. Data shown are mean ± s.d. from triplicate well. The experiments were repeated 3 times. ***P< 0.001, **P<0.01 (Unpaired two-tailed t-test)

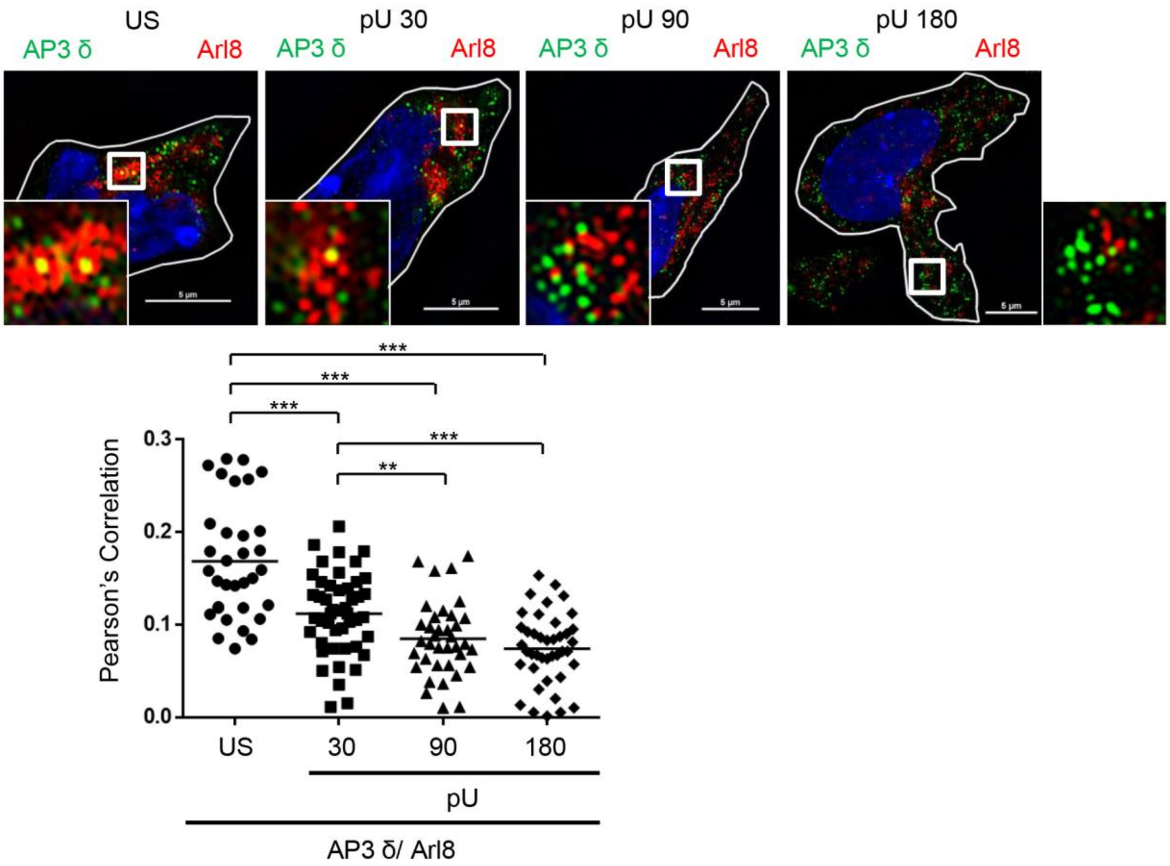

Supplementary Figure 9. Colocalization of Arl8 and AP3 decreases by TLR7 activation in BM-pDCs.

WT BM-pDCs were stimulated or not (US) with polyU at 25 μg/ml for 30 (pU 30), 90 (pU 90), and 180 min (pU 180). pDCs were stained with Abs against AP3 δ (green), and Arl8 (red) as indicated. Nuclei were visualized via DAPI staining (blue). Bottom panel shows statistical analyses of AP3 δ co-localization with Arl8 in each cells (n>32). Scale bar, 5μm. ***P<0.001, **P<0.01 (Unpaired two-tailed t-test)

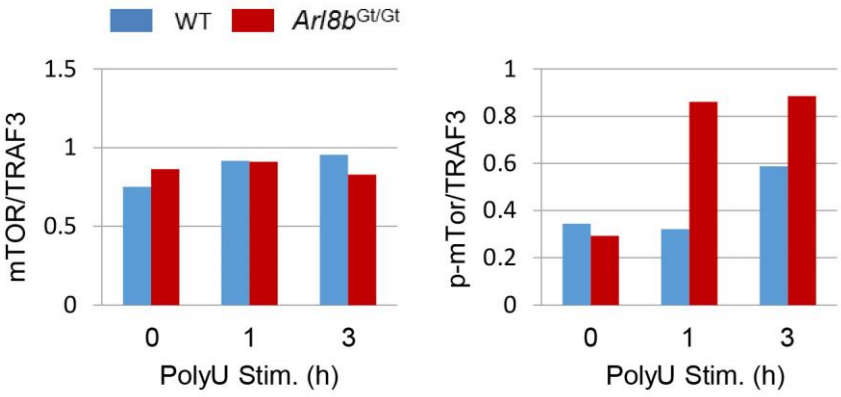

Supplementary Figure 10. Quantification analysis of mTOR and phosphorylated-mTOR associated with TRAF3

The intensity of the signals shown in Fig. 6b was quantified by a software ImageJ to compare WT and *Arl8b*$^{Gt/Gt}$ BM-pDCs in the amount of mTOR associated with immunoprecipitated TRAF3 (right panel) and its phosphorylation induced by polyU stimulation.

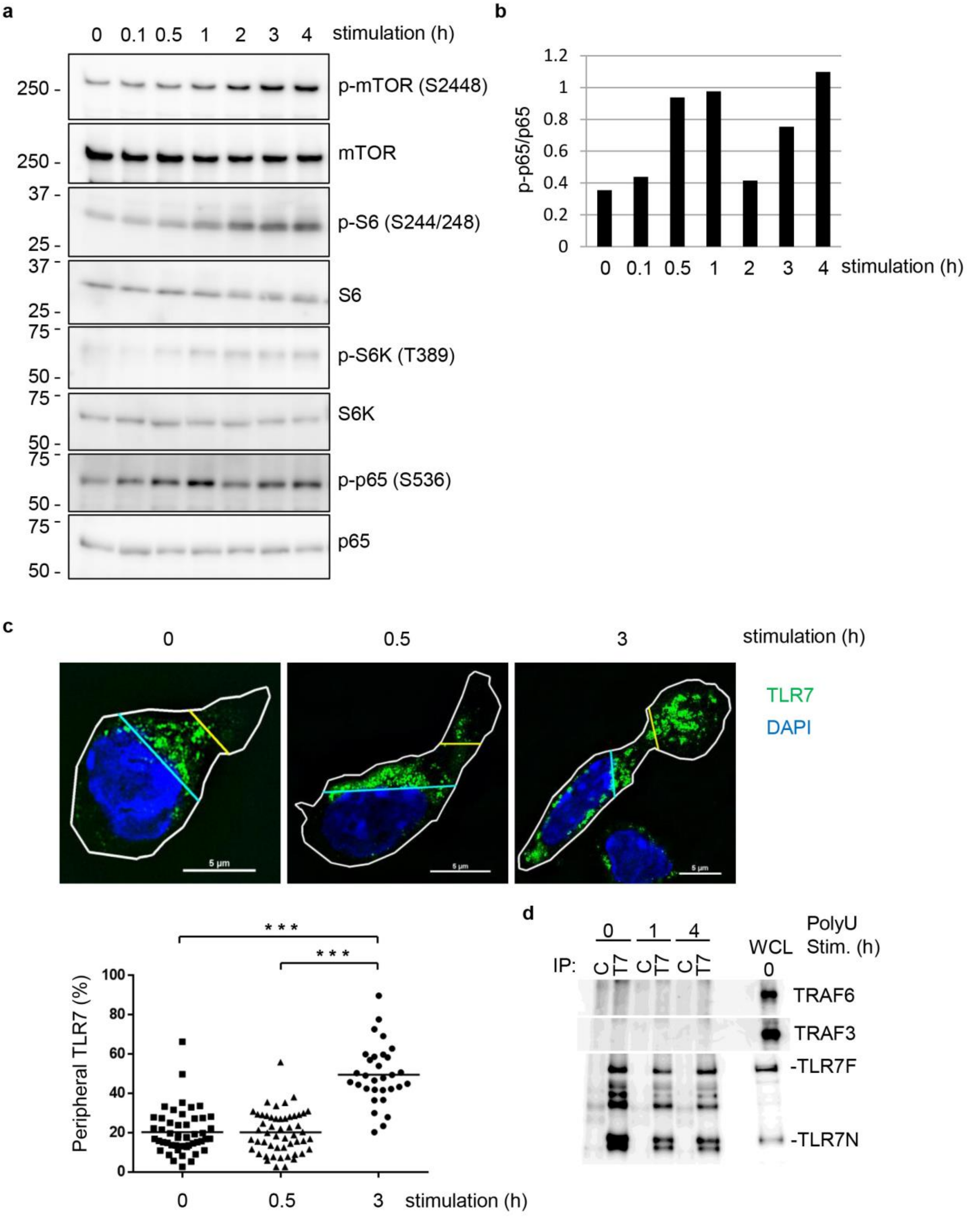

Supplementary Figure 11. TLR7 trafficking correlates with delayed activation of NF-κB and mTORC1

(**a**) WT BM-pDCs were activated with 25 μg/ml polyU for the indicated periods of time. Immunostaining of the indicated signaling molecules in whole cell lysate from WT BM-pDC is shown. (**b**) The ratio of phosphorylated p65 over p65 in (**a**) were quantified by a software ImageJ, and shown. (**c**) WT BM-pDCs were activated (pU) with 25 μg/ml polyU for 0.5 or 3 h. pDCs were stained with Abs against TLR7 (green). Nuclei were visualized via DAPI staining (blue). Bottom panel shows the quantification of peripheral TLR7 as a percentage of the total staining in perinuclear and peripheral regions in each cell (n>30). Scale bar, 5μm. ***P< 0.001 (Unpaired two-tailed t-test). **(d)** WT BM-pDCs was stimulated with 25μg/ml polyU for the indicated times prior to lysis and immunoprecipitation using Ab against TLR7 (T7) or mouse IgG negative control (C) followed by immunostaining of the indicated signaling molecules. Apparent molecular mass is indicated (left). Immunostaining of TRAF6, TRAF3, and TLR7 in whole cell lysate (WCL) is also shown.

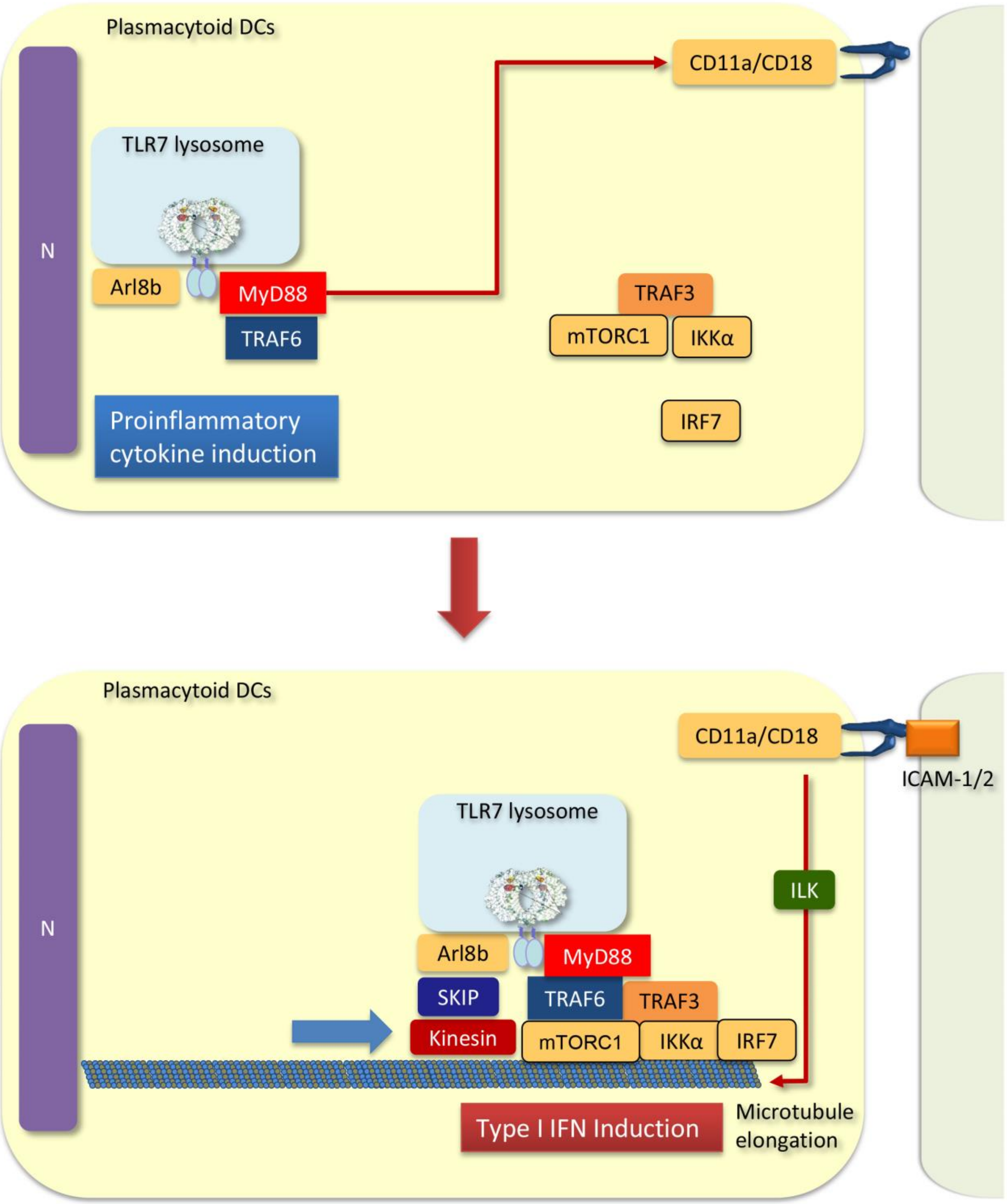

Supplementary Figure 12. Cell adhesion licenses TLR7 to induce IFN-α.

In resting cells, TLR7 is localized to lysosomes around the nuclei (N). TLR7 ligation induces

production of proinflammatory cytokines and promotes cell adhesion through MyD88-dependent signaling pathways (upper panel). Activated CD11a/CD18 induces microtubule elongation through the ILK-dependent signaling pathway (lower panel). TLR7 also activates GTPase Arl8b to link TLR7-containing lysosomes with polymerized microtubule and traffic to cell periphery. Then, TLR7 activates the molecular complex consisting of TRAF3, IKKα, and mTORC1 in the peripheral region to induce IFN-α. IFN-α is produced predominantly in pDC clusters.

Fig.3a

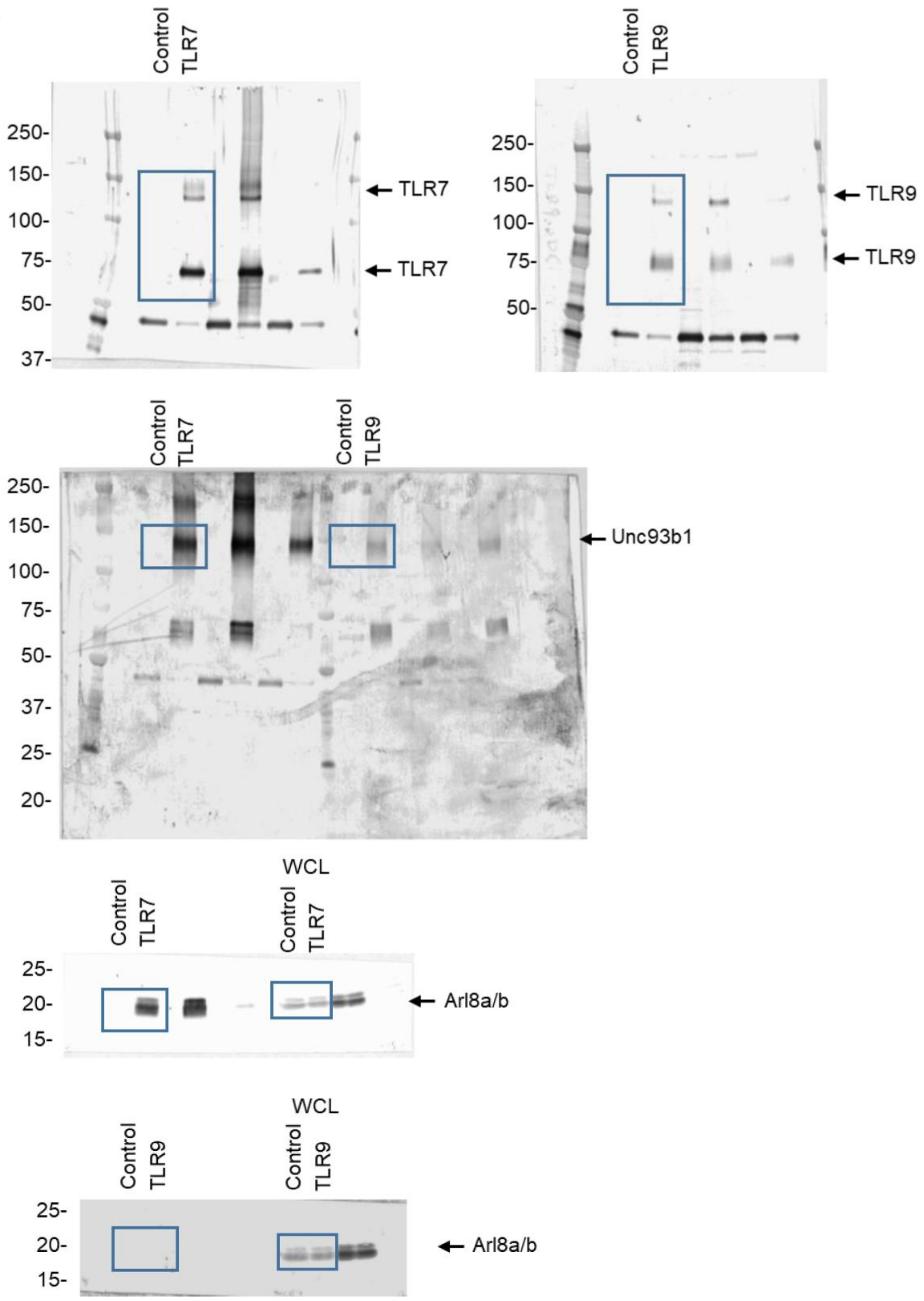

Supplementary Figure 13. Uncropped Western Blot images.

Fig.6a

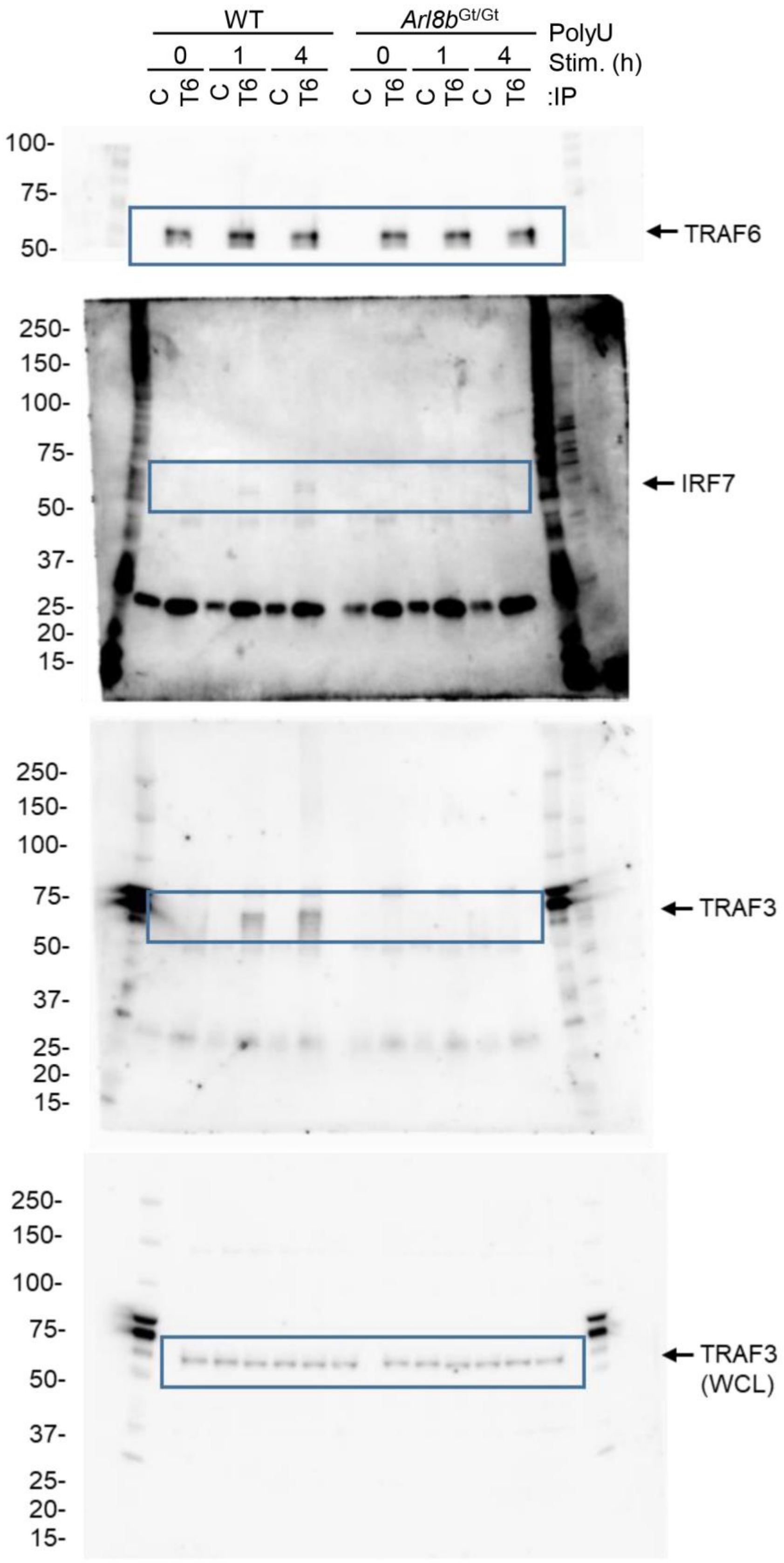
WT
Arl8b^Gt/Gt
PolyU
0 1 4 0 1 4
Stim. (h)
C T6 C T6 C T6 C T6 C T6 C T6
:IP
100-
75-
50-
TRAF6
250-
150-
100-
75-
50-
37-
25-
20-
15-
IRF7
250-
150-
100-
75-
50-
37-
25-
20-
15-
TRAF3
250-
150-
100-
75-
50-
37-
25-
20-
15-
TRAF3 (WCL)

Supplementary Figure 14. Uncropped Western Blot images.

Fig.6b

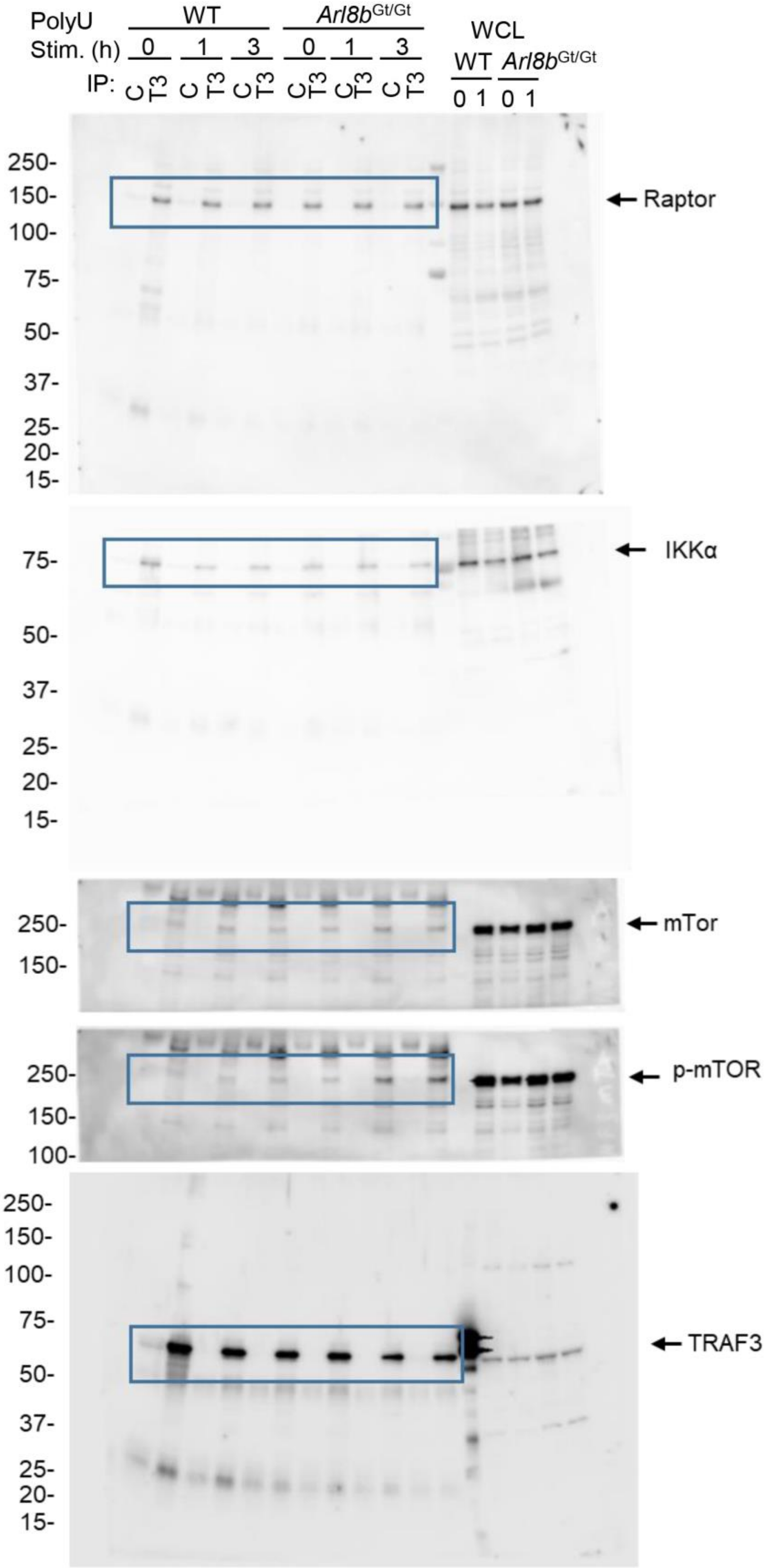
PolyU WT Arl8b^Gt/Gt
Stim. (h) 0 1 3 0 1 3
IP: C T3 C T3 C T3 C T3 C T3 C T3
WCL
WT Arl8b^Gt/Gt
0 1 0 1
250- 150- 100- 75- 50- 37- 25- 20- 15-
Raptor
75- 50- 37- 25- 20- 15-
IKKα
250- 150-
mTor
250- 150- 100-
p-mTOR
250- 150- 100- 75- 50- 37- 25- 20- 15-
TRAF3

Supplementary Figure 15. Uncropped Western Blot images.
